# Gastrointestinal Parasites in Himalayan Musk Deer (*Moschus leucogaster* Hodgson, 1839) and Sympatric Livestock in Gaurishankar Conservation Area, Nepal

**DOI:** 10.1002/vms3.71266

**Published:** 2026-10-13

**Authors:** Rabin Sharma, Narayan Prasad Koju, Aakriti Adhikari, Mahendra Maharjan, Rashila Deshar

**Affiliations:** ^1^ Central Department of Environmental Science Tribhuvan University Kirtipur Nepal; ^2^ Department of Environment Amrit Campus, Tribhuvan University Kathmandu Nepal; ^3^ Center for Postgraduate Studies, Nepal Engineering College Pokhara University Lalitpur Nepal; ^4^ Central Department of Zoology Tribhuvan University Kirtipur Nepal

**Keywords:** cross‐transmission, fecal analysis, habitat overlap, *Muellerius capillaris*, overgrazing, sympatric host

## Abstract

**Background:**

Parasite infections threaten both domesticated and wildlife populations. Shared resources between wildlife and domesticated ruminants increase the risk of cross‐transmission, underscoring the need for infection research. This study aimed to determine the composition, prevalence, and habitat‐dependent patterns of gastrointestinal parasites in endangered Himalayan musk deer (*Moschus leucogaster*) and sympatric livestock (chauri and horses) in Lapchi Valley of Gaurishankar Conservation Area.

**Method:**

Using a cross‐sectional, comparative, systematic, and grazing regime‐based study design along belt transects, a total of 97 fecal samples of Himalayan musk deer (*n* = 67) and livestock (*n* = 30) were collected from livestock‐influenced habitat and natural habitat. A prior sample size calculation was not performed; instead, all accessible musk deer latrines and available livestock encountered during the survey period were sampled. Parasite taxa were identified based on morphological characteristics.

**Results:**

The analysis revealed an overall prevalence of 94.8%. Twelve parasites were identified, including two protozoans (*Eimeria* sp. and *Entamoeba* sp.), eight nematodes (ascarid eggs, *Bunostomum* sp., *Capillaria* sp., *Dictyocaulus* sp., *Muellerius capillaris*, *Oxyuris* sp., Strongylida larvae, and *Trichostrongylus* sp.), one cestode (*Moniezia* sp.), and one trematode (*Paramphistomum* sp.). Among musk deer samples (*n* = 67), 92.5% were positive, with nematodes being most prevalent. Six parasite species were common to both livestock and musk deer, indicating potential cross‐transmission. *Muellerius capillaris* was most prevalent in musk deer (85.1%), while Strongylida larvae were most prevalent in livestock (56.7%). Higher prevalence in musk deer was observed in livestock‐influenced areas than in natural habitats.

**Conclusions:**

Although parasite compositions showed distinct clustering by host, the presence of six shared species underscores the risk of spillover. These findings accentuate the need for integrated parasite management strategies that minimise livestock‐wildlife contact as a critical component of conservation planning in protected areas.

## Graphical abstract

This research shows high gastrointestinal parasitic infections in the endangered Himalayan musk deer, indicating a significant risk of cross‐transmission from sympatric livestock (chauri and horses). Moreover, distinct host‐specific parasite dominance and habitat‐dependent infection patterns accentuate the need for minimising livestock‐wildlife contact in conservation planning to safeguard wild musk deer populations.

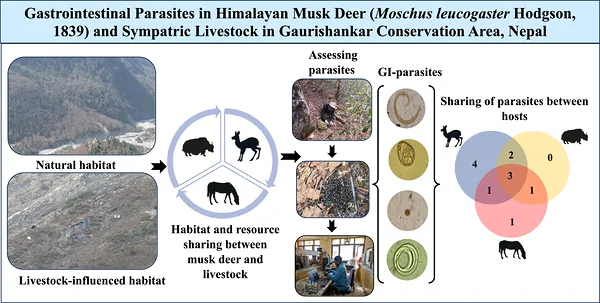

## 1 Introduction

The musk deer is an ecologically and economically viable Himalayan ungulate belonging to the family Moschidae, comprising a single genus, *Moschus*, distributed across countries such as Nepal, India, China, Bhutan, Myanmar, Pakistan, and Afghanistan (Green 1986; Groves and Grubb 1987). Nepal provides suitable habitats for musk deer, with an estimated potential range of 10,000 km^2^ across several protected areas. It is found across the Api Nampa Conservation Area, Khaptad National Park, Rara National Park, Shey Phoksundo National Park, Annapurna Conservation Area, Gaurishankar Conservation Area, and others (Aryal et al. 2010; Aryal and Subedi 2011; Green 1986; Jnawali et al. 2011; Shrestha et al. 2018). Musk deer is classified as an ‘Endangered’ species by the International Union for Conservation of Nature (IUCN) and listed in Appendix I of the Convention on International Trade in Endangered Species of Wild Fauna and Flora (CITES) (IUCN, 2009; Jnawali et al. 2011). Its populations face significant threats from degradation of habitat, illegal poaching for musk pods, livestock overgrazing, and disease (Green 1986; Jnawali et al. 2011; Mishra et al. 2004).

Disease occurrence results from various factors that facilitate parasite transmission and the emergence of novel diseases (Streicker et al. 2010). Until the 1970s, the effects of diseases on wildlife were mostly overlooked. It was widely believed by the general public, wildlife managers, and even academics that wildlife possessed strong immune systems capable of coping with infections (Joseph et al. 2013). However, in recent years, the broader impact of infectious diseases on global biodiversity conservation has become more widely recognised and gained attention from researchers and conservation organisations (Smith 2022).

While wild ruminants harbour diverse gastrointestinal parasites asymptomatically, heavy parasite burdens can adversely affect host health, survival, and reproductive success (Hu et al. 2018a). Under high population densities, nutritional deprivation, or close contact with domestic livestock, visible symptoms of parasitic disease can emerge. In these severe cases, pathogenic infections may lead to tissue damage, blood loss, reduced fertility, and congenital malformations, ultimately reducing physiological and immune performance of endangered species like the musk deer (Li et al. 2018; Thawait et al. 2014). At the same time, in sympatric domestic breeding systems, such parasite infections pose a threat to livestock health and productivity (Biffa et al. 2006).

Gastrointestinal diseases are particularly common in musk deer, often leading to high mortality rates (Achhami et al. 2016; Hu et al. 2018a; Hu et al. 2018b; Li et al. 2017; Li et al. 2018; Xu et al. 2014). Approximately 35% of deaths are attributed to coccidian protozoan parasites that cause coccidiosis, which damage the host's intestinal epithelial cells and increase susceptibility to bacterial invasion, often leading to fatal diarrhoea (Yan et al. 2016). Beyond diarrhoea, musk deer experience imbalances in gut macrobacteria, making them more vulnerable to small environmental stressors (Hu et al. 2017; Li et al. 2018).

*Enterocytozoon bieneusi* was detected through molecular studies, with an infection rate of 6% in captive alpine musk deer in China. The identified genotypes are common to both humans and animals, indicating potential zoonotic transmission (Zhao et al. 2022). *Giardia duodenalis* and *Enterocytozoon bieneusi* were reported for the first time in forest musk deer in China by Song et al. (2018). This highlights the potential role of musk deer as reservoirs in the forest environment as well. Studies in Langtang National Park, Nepal, have documented several gastrointestinal parasites in musk deer, including *Eimeria* sp., *Ascaris* spp., *Strongyloides* spp., *Trichuris* spp., *Moniezia* spp., and *Paramphistomum* spp. (Achhami et al. 2016).

Habitat preferences and grazing practices of wild ungulates and livestock are major factors influencing parasite transmission within and between species (Ezenwa 2003). Parasites rely on their hosts for nourishment and shelter, and shared habitats facilitate cross‐species transmission. The occurrence of *Eimeria* spp. in both musk deer and chauri, a hybrid of yak and cow (hereafter chauri), in Langtang National Park, suggests possible mutual transmission between livestock and musk deer (Achhami et al. 2016; Sharma and Achhami 2022).

The local communities in the study area have a semi‐nomadic lifestyle and are primarily engaged in chauri herding. Due to limited pasture availability, they move seasonally to higher elevations, establishing scattered camps across the alpine meadows. The sharing of grazing land between domesticated ruminants and wildlife increases the risk of parasite transmission and poses a threat to endangered species. Parasitic infections in wildlife can affect both individuals and populations, making them a crucial issue for the conservation of threatened species (Thompson et al. 2009). Early prediction and monitoring are essential to prevent parasite transmission between domestic and wild hosts. Thus, this study aims to assess gastrointestinal parasite prevalence in Himalayan musk deer and sympatric livestock (chauri and horse) in the Lapchi Valley of Gaurishankar Conservation Area, Nepal, to better understand parasite dynamics and inform conservation efforts.

## 2 Materials and Methods

### 2.1 Study Area

The study was conducted in Lapchi Valley, located within the Gaurishankar Conservation Area (GCA) of Nepal (Figure 1). Lapchi Valley lies within the geographic coordinates of 86°10'32.55‘’E to 86°29'9.48‘’E longitude and 28°20'13.29‘’N to 28°21'54.50‘’N latitude, with an elevation range of 2600 to 4950 m above sea level (m asl) (NTNC, 2013). The valley is bordered by China on the east, west, and north sides and is situated at the base of the Lapchi Khang mountain range. Lapchi Valley holds significant cultural and religious importance as a pilgrimage site for Tibetan Buddhists. The GCA is characterised by rich floral and faunal diversity. According to the National Trust for Natural Conservation, the GCA supports 695 plant species, including flowering, non‐flowering, medicinal, and aromatic plants. The faunal diversity includes 71 mammal species, 24 fish species, 12 amphibian species, 27 reptile species, and 252 bird species (NTNC, 2013). Himalayan musk deer are primarily found at higher altitudes within mixed forests and scrubland areas, particularly in the Lapchi Valley of GCA (Koju et al. 2021; Shrestha et al. 2018). In Lapchi Valley, the local Sherpa community of Lapchi Village (39 households) primarily relies on a semi‐nomadic lifestyle and seasonal transhumance for their livelihood. Their economy primarily depends on chauri husbandry, along with horse rearing used for transport, trade, and income.

**FIGURE 1:**
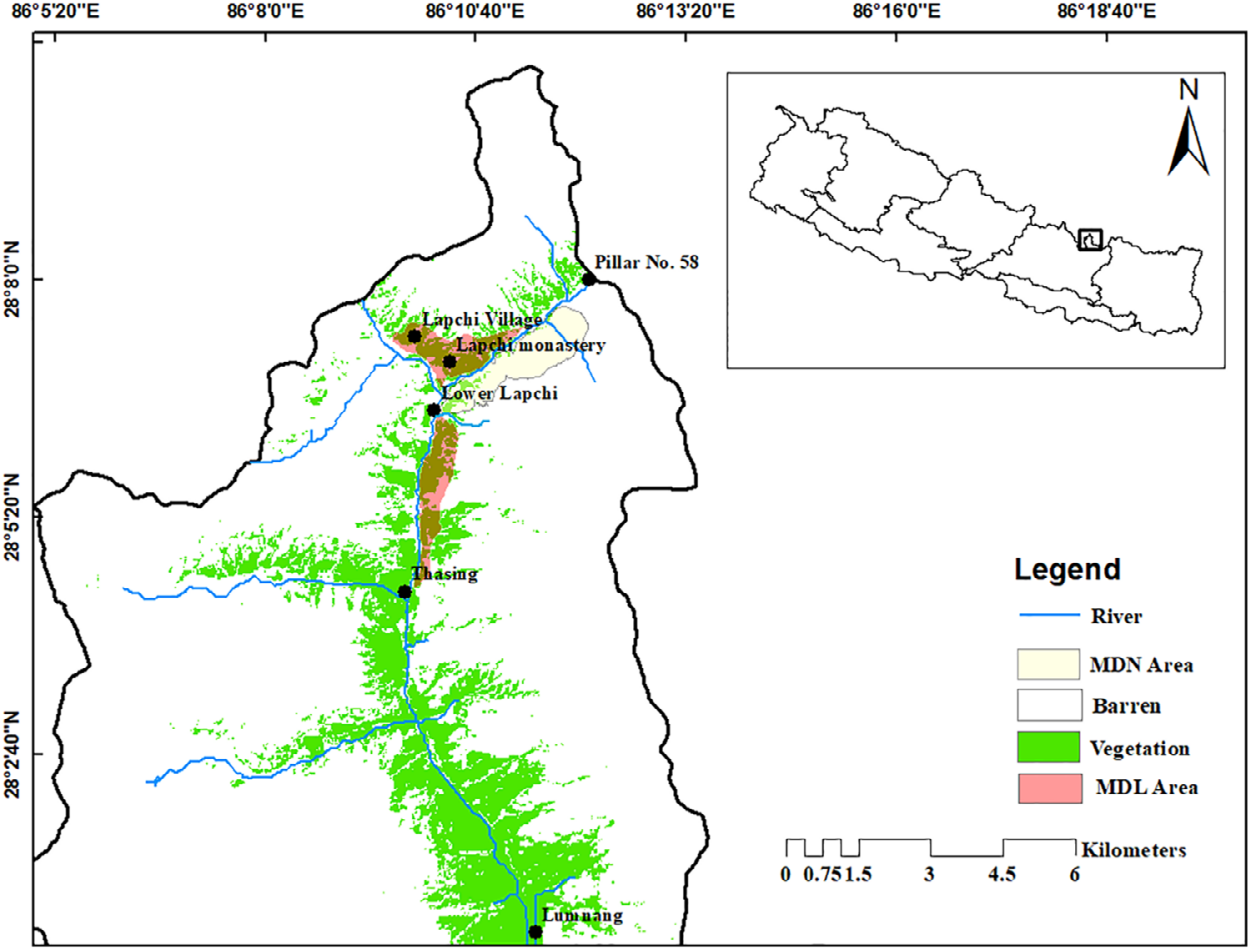
Sampling locations in the Lapchi Valley, Gaurishankar Conservation Area, Nepal.

### 2.2 Study Design

A cross‐sectional, comparative, systematic, and grazing regime‐based study design was used to evaluate the gastrointestinal parasite communities of Himalayan musk deer (*Moschus leucogaster*) and sympatric livestock (horses and chauri) in Lapchi Valley. The study evaluated differences in gastrointestinal parasite prevalence, diversity, and community composition in musk deer and sympatric livestock. Further, it assessed the potential impact of habitats with different levels of livestock influence on parasite communities.

This study compared four host groups: musk deer inhabiting natural habitats (MDN), musk deer inhabiting livestock‐influenced areas (MDL), horses, and chauri. The two musk deer groups were defined based on habitat characteristics and the presence or absence of livestock activity. Horses and chauri were included as sympatric domestic hosts to characterise their gastrointestinal parasite communities and compare them with the parasite communities detected in the musk deer.

The study followed a comparative observational design in which no experimental manipulation of host populations or study sites was performed. Fresh fecal samples were systematically collected for each host group from MDL and MDN and examined for gastrointestinal parasites using standard parasitological techniques. Thus, the study design enabled an assessment of the association between livestock influence and gastrointestinal parasite communities of musk deer while also providing information on parasite communities occurring in sympatric domestic hosts. This comparative approach was used to determine whether MDL showed differences in gastrointestinal parasite communities compared with MDN.

### 2.3 Study Sites

The MDN site lies at an average elevation of 3880 m asl, southeast of Lapchi Village, extending from below Border Pillar No. 58 to Lower Lapchi. The area is separated from the MDL site by the Lapchekhun River, a glacier‐fed river originating near the Nepal‐Tibet border. The river, together with rugged terrain and steep cliffs, forms a natural barrier that limits livestock access to the MDN site. This site consists of subalpine *Rhododendron anthopogon* and Himalayan birch (*Betula utilis*) forest. Consequently, this relatively undisturbed area experiences minimal human activity and negligible interactions between musk deer and livestock.

The MDL site comprises two sections: the first section is located between Lower Lapchi and Thasing, southeast of Lapchi Village at 3270 to 3780 m asl, whereas the second section surrounds Lapchi Village at 3831 to 4038 m asl.

The first section comprises subalpine mixed‐conifer forest interspersed with alpine meadows, with dense moss‐covered vegetation dominated by Himalayan fir (*Abies spectabilis), Rhododendron campaunulatum*, and *Rhododendron barbatum*, with scattered individuals of *Tsuga dumosa* and *Taxus* spp. The site is separated from MDN by a deep gorge that restricts livestock movement. The terrain is comparatively flat and is heavily used by local agro‐pastoralists for livestock grazing, and includes several active chauri sheds. During winter, livestock are moved from upper pastures along this route, passing through Thasing toward Lumnang. This seasonal transhumance movement creates high livestock activity and increased contact between livestock and musk deer within shared areas.

The second section, surrounding Lapchi Village, consists of fragmented shrubland and alpine pastures, with scattered juniper thickets. This section includes the renowned meditation caves of Jetsun Milarepa, located around Chora Gephel Ling. The section is characterised by large boulders and rocky terrain, with trails leading to the pilgrimage site and associated meditation caves. The area is inhabited by local Sherpa communities who rely on livestock‐rearing for their livelihood and allow their livestock to graze freely across the area. During summer, livestock are moved from the village to high‐altitude pastures along the Nepal‐Tibet border. Consequently, the area experiences high levels of human disturbance associated with livestock management and local resource extraction, including tree lopping, logging, foot trail utilisation, and forest fires. These combined activities increase the potential for contact between livestock and musk deer.

### 2.4 Sample Collection and Parasite Identification

A cross‐sectional, comparative grazing regime‐based study design with systematic sampling was used to collect fecal samples from Himalayan musk deer and sympatric livestock across MDL and MDN sites. Within each study site, belt transects (100 m × 100 m) were established, and sampling points were systematically placed at 300–400 m intervals along each transect to minimise the risk of pseudo‐replication (Achhami et al. 2016). During field surveys, only fresh, greenish, moist fecal samples were collected from independent latrine sites. Fecal samples were distinguished based on their morphological characteristics and field context. Musk deer pellets were identified by their small, uniform, oval‐to‐cylindrical shape and presence of localised latrines (Green 1986; Kattel, 1992). Additionally, horse and chauri feces were differentiated based on morphological features associated with their digestive anatomy, with horses producing large, fibrous, rounded boluses and chauri depositing soft, amorphous ruminant dung pats.

An a priori sample size calculation was not performed. Instead, the sample size was determined by field availability, accessibility, and logistical constraints. Consequently, all accessible musk deer latrines and available livestock encountered within the predefined survey plots were sampled during the study period to maximise sample coverage. A total of 97 fresh fecal samples were collected, comprising 67 samples from musk deer (MDL = 32; MDN = 35) and 30 samples from livestock (chauri = 15; horses = 15). All samples were collected in 30 mL sterile vials containing 10 mL of 2.5% potassium dichromate solution. The samples were immediately transported to the laboratory of the Central Department of Environmental Science, Tribhuvan University, Kathmandu, and maintained at 4°C until analysis, which was conducted within one month of collection.

Fecal samples were analysed using the saline wet mount method. Approximately 2 g of each fecal sample was emulsified with normal saline and stained with Lugol's iodine. A 0.10 mL aliquot of the suspension was placed on a microscope slide, covered with a coverslip, and examined under a compound light microscope (BIO2) at 100× and 400× magnifications. To improve the detection of low abundance of eggs and oocysts in fecal samples, concentration methods such as Stoll's flotation method (Stoll 1930) and Young et al.’s modified sedimentation method (Young et al. 1979) were applied. Suspected parasitic structures seen under a microscope were photographed using a digital microscope camera (Model: BLACKL. 5000, S/N: 98768) operated via the BEL Eurisko application. The length and other relevant dimensions of parasitic stages were digitally measured using the BEL Eurisko application for further morphological confirmation. Parasite identification was performed at the genus level based on morphological keys and descriptions (Byford et al. 2007; Foreyt 2013; Soulsby 1982).

### 2.5 Data Analysis

Parasite prevalence was calculated for each parasite taxon and host species as the proportion of positive fecal samples relative to the total number of samples examined. To assess multivariate patterns in parasite prevalence among host groups, an ordination analysis examined the community structures, followed by Permutational Multivariate Analysis of Variance (PERMANOVA) with 999 permutations to test the statistical significance of observed differences. This analysis utilised parasite presence/absence (binary) data as the multivariate response. The Bray–Curtis dissimilarity index was used to calculate the distance matrix between samples. It is appropriate for binary presence‐absence data and emphasises shared species while ignoring joint absences. All statistical analyses were performed using R version 4.2.3 with the vegan package to ensure robust inference of parasite species differences among host types.

## 3 Results

### 3.1. Prevalence and Sharing of Gastrointestinal Parasites Among Hosts

Out of 97 samples examined, 92 samples (94.8%) were found positive for one or more specific gastrointestinal (GI) parasites in the Lapchi Valley of Gaurishankar Conservation Area. Overall, 12 species of GI parasites occurred across three host types (Table 1). Protozoan parasites included *Eimeria* sp. and *Entamoeba* sp., whereas nematodes consisted of ascarid eggs, *Bunostomum* sp., *Capillaria* sp., *Dictyocaulus* sp., *Muellerius capillaris*, *Oxyuris* sp., Strongylida larvae, and *Trichostrongylus* sp. Additionally, the cestode (*Moniezia* sp.) and the trematode (*Paramphistomum* sp.) represented the flatworm infections. Collectively, eight gastrointestinal parasites infected sympatric livestock (chauri and horse), including the protozoan (*Eimeria* sp.); the nematode (ascarid eggs, *Dictyocaulus* sp., Strongylida larvae, and *Trichostrongylus* sp.); the cestode (*Moniezia* sp.); and the trematode (*Paramphistomum* sp.). Among these, the nematode (*Oxyuris* sp.) occurred exclusively in horses. Overall, Strongylida larvae dominated livestock infections, with a prevalence of 56.7%. Individually, this parasite exhibited a higher prevalence in horses (66.7%) than in chauri (46.7%).

**TABLE 1:** Prevalence of gastrointestinal parasites in Himalayan musk deer and sympatric livestock (chauri and horse).

Taxonomic groups	Name of parasites	Musk deer (%) (*n* = 67)	Chauri (%) (*n* = 15)	Horse (%) (*n* = 15)
Protozoa	*Entamoeba* sp.	13.4	−	−
	*Eimeria* sp.	7.5	26.7	13.3
	Ascarid eggs	35.8	60	40
	*Bunostomum* sp.	11.9	−	−
	*Capillaria* sp.	5.9	−	−
Nematode	*Dictyocaulus* sp.	8.9	−	20
	*Muellerius capillaris*	85.1	−	−
	Strongylida larvae	7.5	46.7	66.7
	*Oxyuris* sp.	−	−	13.3
	*Trichostrongylus* sp.	−	33.3	46.7
Cestode	*Moniezia* sp.	2.9	26.7	−
Trematode	*Paramphistomum* sp.	11.9	13.3	−

The sharing of parasites between three hosts shows that one protozoan (*Eimeria* spp.) and two nematodes (ascarid eggs and Strongylida larvae) infected musk deer, chauri, and horses. In contrast, one protozoan (*Entamoeba* sp.) and three nematodes (*Bunostomum* sp., *Capillaria* sp., and *Muellerius capillaris*) occurred exclusively in musk deer. Cross‐species occurrence varied among the remaining taxa, including the nematode (*Dictyocaulus* sp.) that infected both musk deer and horses, while the cestode (*Moniezia* sp.) and the trematode (*Paramphistomum* sp.) infected only musk deer and chauri. Finally, the nematode (*Trichostrongylus* sp.) infected both chauri and horses (Figure 2).

**FIGURE 2:**
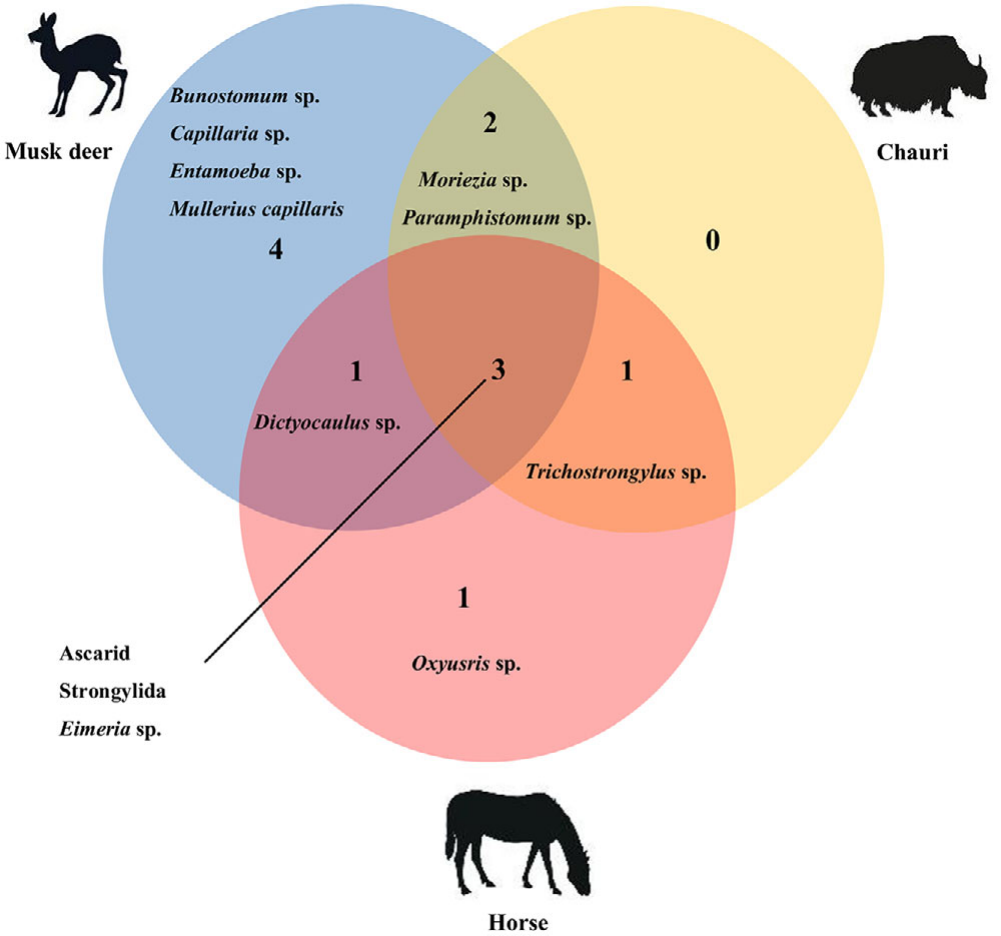
Venn diagram showing the distribution of gastrointestinal parasites among musk deer, chauri, and horse with common species in overlapping sections and uncommon species in non‐overlapping sections.

### 3.2. Prevalence of Gastrointestinal Parasite Infection in Musk Deer

Out of 67 musk deer pellet samples examined, 92.5% were infected with one or more species of gastrointestinal parasites. Nematodes were the most prevalent group that occurred in the majority of the samples, followed by protozoans, trematodes, and cestodes with lower prevalence. Out of six nematodes, *Muellerius capillaris* exhibited the highest prevalence of 85.1% (Table 1). Other nematodes identified include ascarid eggs, *Bunostomum* sp., *Capillaria* sp., *Dictyocaulus* sp., and Strongylida larvae. Cestodes were represented by *Moniezia* sp., with a prevalence of 2.9%, while a single trematode (*Paramphistomum* sp.) had a prevalence of 11.9%.

Protozoan parasites occurred in 19.4% of the musk deer pellets, with *Entamoeba* sp. and *Eimeria* sp. prevalences of 13.4% and 7.5%, respectively. In contrast to these relatively low protozoan rates, the results indicate a much higher presence of nematode infections among the populations of musk deer in the Lapchi Valley, where *Muellerius capillaris* constitutes the most prevalent parasite species.

### 3.3. Habitat‐Wise Variation of Gastrointestinal Parasites in Musk Deer

A comparison of the prevalence of gastrointestinal parasites of musk deer in livestock‐influenced areas (MDL) and natural habitats (MDN) revealed very similar infection rates, with overall parasite prevalence of 93.7% and 91.4%, respectively. A higher prevalence of protozoan infection was observed in the MDL (25.0%) than in the MDN (14.3%). Among protozoans, *Entamoeba* sp. showed a greater prevalence in the MDL (18.8%) compared to the MDN (8.5%). In contrast, *Eimeria* sp. exhibited less variation between habitats, with a prevalence rate of 9.4% in musk deer from the MDL and 5.7% from the MDN (Figure 3).

**FIGURE 3:**
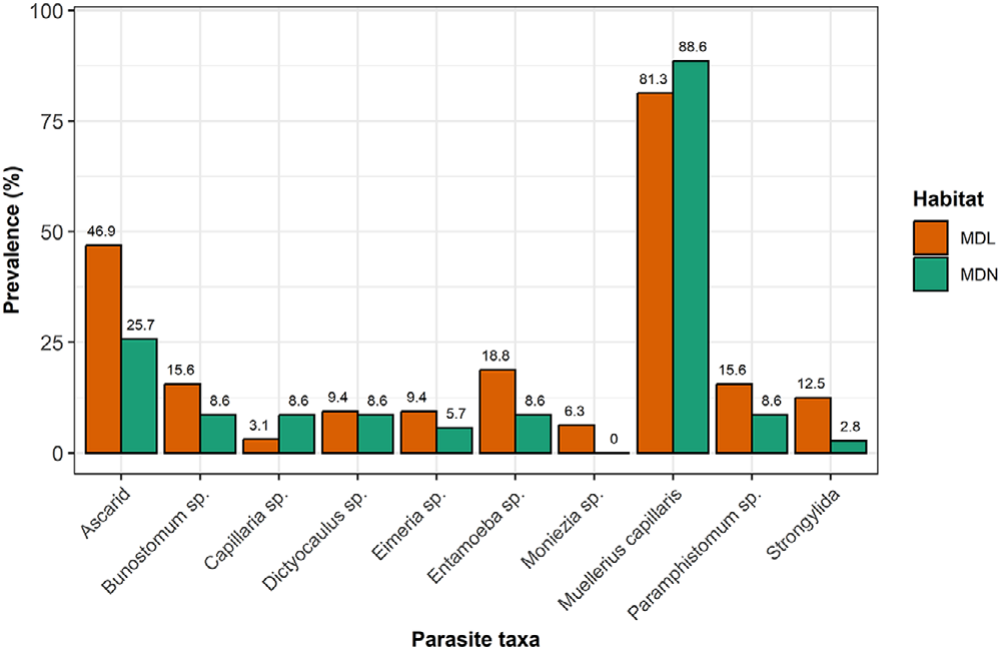
Prevalence of gastrointestinal parasites in musk deer.

Nematodes comprising ascarid eggs, *Bunostomum* sp., *Capillaria* sp., *Dictyocaulus* sp., and *Muellerius capillaris* were commonly present in both habitats. The prevalence of ascarid eggs, *Bunostomum* sp., and Strongylida larvae was higher in the MDL. Conversely, a higher prevalence of *Muellerius capillaris* and *Capillaria* sp. was observed in the MDN. The cestode *Moniezia* sp.) showed a prevalence of 6.3% in the MDL. Similarly, the prevalence of the trematode (*Paramphistomum* sp.) was lower in the MDN compared to the MDL.

### 3.4. Group Influence on Parasite Community Composition

Principal coordinates analysis (PCoA) based on Bray–Curtis dissimilarity revealed distinct variations in gastrointestinal parasite community composition among the different host groups (chauri, horse, MDL, and MDN) (Figure 4). Horse and chauri samples clustered closely together, exhibiting relatively similar parasite communities. Musk deer samples (MDL and MDN) showed considerable overlap, indicating that habitat difference alone may not strongly separate parasite communities. However, MDL samples showed partial overlap with livestock patterns, suggesting potential parasite spillover between them. PERMANOVA further validated these patterns, showing that the host group explained 38.2% of the total variation in parasite community composition (*F*  = 18.13, *p*  = 0.001), confirming statistically significant clustering.

**FIGURE 4:**
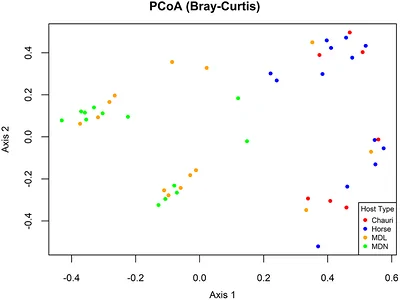
Composition of gastrointestinal parasite communities in chauri (red), horse (blue), MDL (orange), and MDN (green). The clustering of points by colour indicates that parasite communities are more similar within host groups and distinct between them. The analysis is based on presence‐absence data of 12 parasite species.

## 4 Discussion

This study showed a high prevalence of gastrointestinal (GI) parasites among musk deer and sympatric livestock (chauri and horse) around Lapchi Valley, with 94.8% of samples containing one or multiple GI parasites. Musk deer were infected with two protozoans (*Eimeria* sp. and *Entamoeba* sp.), six nematodes (ascarid eggs, *Bunostomum* sp., *Capillaria* sp., *Dictyocaulus* sp., *Muellerius capillaris*, and Strongylida larvae), one cestode (*Moniezia* sp.), and one trematode (*Paramphistomum* sp.). These findings are consistent with previous studies which documented high parasite prevalence in both wild and captive musk deer (Achhami et al. 2016; Hu et al. 2018a).

Out of 12 parasite species identified, one parasite was found in both musk deer and horse, two parasites were found in musk deer and chauri, and three were found in all three host species. Protozoan infections were limited to *Eimeria* sp. and *Entamoeba* sp., while nematode parasites included ascarid eggs, *Dictyocaulus* sp., Strongylida larvae, and *Trichostrongylus* sp. Additionally, cestode (*Moniezia* sp.) and trematode (*Paramphistomum* sp.) infections were also detected in all host species. These findings support the hypothesis that habitat overlap and resource sharing between musk deer and livestock might help cross‐species transmission of these parasites, a similar phenomenon previously documented in similar ecosystems (Achhami et al. 2016; Sharma and Achhami 2022). A comprehensive review by Walker and Morgan (2014) reported substantial overlap and potential for cross‐species transmission between wild and domestic ungulates.

The present study showed gastrointestinal parasite prevalence (92.5%) in musk deer, while this prevalence is comparable to the 88.9% reported in musk deer samples from Langtang National Park (Achhami et al. 2016). Among musk deer samples, protozoan parasites were detected in 19.4%, with *Eimeria* sp. contributing a prevalence of 7.2%. This prevalence is considerably lower than that reported by Gao et al. (2021), reporting 65% prevalence of *Eimeria* spp. in captive forest musk deer from multiple breeding centres in China and identified six new species. Such differences in the prevalence rates may be attributed to variations in environmental and management factors, including soil moisture, sanitation, and climatic conditions (Nunn et al. 2005).

*Eimeria* spp. are recognised as a primary cause of mortality in small ruminants younger than four months old (Harwood 2006). In this study, 7.5% prevalence of *Eimeria* spp. was detected in musk deer, which closely matches records from white‐tailed deer in Georgia (Kingston and Stabler 1978). These coccidia parasites damage the host's intestinal epithelial cells, which increases the flow of tissue fluid into the intestinal lumen, making them more vulnerable to secondary bacterial invasions. This cellular infection includes watery diarrhoea, which can progress to severe enteritis and bloody diarrhoea in infected hosts (Jithendran 2002).

Among the various nematode taxa identified in musk deer, *Muellerius capillaris* exhibited the highest prevalence (85.1%). This finding is consistent with previous studies reporting *M. capillaris* as a common lungworm infecting wild and domestic small ruminants (Foreyt et al. 2009; Kowal et al. 2016). For instance, Ezenwa et al. (2010) documented a 100% prevalence of *M. capillaris* in wild sheep populations, and it is widely recognised as a common parasite in these hosts (Ezenwa et al. 2010; Foreyt et al. 2009). The presence of *M. capillaris* in blue sheep (*Pseudois nayaur*) has been reported in India (Karikalan et al. 2020), and similar pathological findings associated with lungworm infections have been observed in musk deer (Laha and Harbola 2001).

Lapchi Valley also serves as a prime habitat of blue sheep; this habitat overlap likely facilitates parasite transmission among sympatric wild ungulates. Supporting these studies, Chartier and Reche (1992) and Doumenc et al. (2004) reported a high occurrence of *M. capillaris* infection rates ranging from 85% to 95% in domestic small ungulates. In contrast, the prevalence of *Dictyocaulus* sp. was only 8.9%, markedly lower than that of *M. capillaris*, likely due to differences in their life cycles. The high prevalence of *M. capillaris* can be partly attributed to its broad range of intermediate hosts, ‘terrestrial molluscs’, and the larvae's ability to overwinter within these molluscs, ensuring year‐round transmission (Taylor et al. 2007).

The first‐stage larvae can survive for months in fecal pellets, and the infective third‐stage larvae persist for the lifetime of the mollusc intermediate host, reinforcing endemicity. Free‐living larvae of *Dictyocaulus* sp. depend heavily on favourable humidity and temperature conditions for survival and development, which may limit their prevalence in the climate of the region (Terefe et al. 2013).

Similarly, the present study found that ascarid egg prevalence in livestock was twofold higher than in musk deer. According to Hagel and Giusti (2010), the robust outer shell of ascarid eggs enables them to remain viable in the environment for up to 15 years, potentially leading to symptoms such as diarrhoea, malnutrition, and intestinal obstruction in infected hosts. The prevalence rate of parasites in livestock of the study area was much higher than in musk deer, and infections of Strongylida larvae were also detected in livestock. Among the identified parasites in the horses within the study area, the prevalence rates of Strongylida larvae and *Dictyocaulus* sp. were similar to those reported from Mustang District, Nepal (Devkota et al. 2021).

Regarding the cestode parasite, *Moniezia* sp. was found in chauri and musk deer, with a prevalence of 2.9% and 26.4% in musk deer and chauri, respectively. Notably, *Moniezia* sp. was found only in musk deer from livestock‐influenced areas, suggesting potential for interspecies infection. Although *Moniezia* sp. infections are typically asymptomatic and considered harmless, even with high tapeworm burdens, severe infection can lead to diarrhoea, weight loss, and intestinal obstruction (Agrawal et al. 2024).

*Paramphistomum* sp., a trematode, was found at very low prevalence in both chauri and musk deer. *Paramphistomum* sp. prevalence in each host was found to be much lower than that of the other identified parasites. The level and severity of trematodes are influenced by environmental factors that impact the availability, development, and survival of intermediate hosts (Koprivnikar et al. 2006). Numerous species of *Paramphistomum* spp. have been reported from domestic animals and have been identified from the rectum and rumen of ruminants, and some of these species can even be found in humans and pigs' large intestines (Soulsby 1982).

Although parasite communities showed distinct clustering, livestock and musk deer shared 6 of the 12 parasites in common. This finding indicates the presence of core parasites common to both domestic and wild hosts, a pattern commonly observed in multi‐host systems (Pilosof et al. 2015; Walker et al. 2017). However, differences in parasite community composition and the presence of host‐specific species contribute to the separation observed in the ordination analysis (Krasnov et al. 2006; Poulin 2011).

Previous research and reviews have demonstrated that while sympatric host species often harbour overlapping parasite infections, variation in parasite communities arises due to host specificity, ecological factors, and habitat diversity (Freeland 1983; Krasnov et al. 2006). Despite differences in habitat quality, the parasite community of musk deer exhibits minimal variation across habitats. This suggests that local ecological variation and varying levels of livestock influence do not substantially affect the composition and diversity of gastrointestinal parasites and is supported by the study of Beaumelle et al. (2021). This pattern may facilitate the transmission of parasites across common feeding areas (Pettorelli et al. 2003; Portanier et al. 2019).

## 5 Conclusion

This study reveals a high prevalence of gastrointestinal parasites (94.8%) among Himalayan musk deer, chauri, and horse in Lapchi Valley of Gaurishankar Conservation Area, indicating a significant prevalence of parasitic infections. Identified parasites include two protozoans, eight nematodes, one cestode, and one trematode based on the morphological characteristics. Six parasites were found in both livestock and musk deer, indicating the existence of common parasites. However, distinct host‐specific infection patterns were observed, with *Muellerius capillaris*, *Oxyuris* sp., and *Trichostrongylus sp*. in musk deer, horse, and livestock, respectively. Furthermore, *Moniezia* sp. in musk deer was limited to the livestock influence area. Although parasite infections showed distinct clustering by host, out of 12 parasite species identified, the presence of 6 shared species suggests a potential risk of spillover between musk deer and livestock. In conclusion, our study provides an important ecological baseline for a remote and understudied geographic region, showing that musk deer face high baseline parasite pressures regardless of livestock influence. However, livestock presence clearly alters the composition of specific parasite taxa. While our findings rely on morphological identification, future research should integrate molecular tools to achieve species‐level resolution. Using both molecular and morphological analyses will be key to tracking cross‐species parasite transmission pathways, contributing to the development of better plans to protect this endangered ungulate.

## Author Contributions

**Rabin Sharma**: conceptualization, methodology, software, data curation, investigation, formal analysis, funding acquisition, visualisation, writing – review and editing, writing – original draft, resources. **Narayan Prasad Koju**: conceptualization, methodology, supervision, investigation. **Aakriti Adhikari**: writing – review and editing, formal analysis, data curation. **Mahendra Maharjan**: data curation, validation. **Rashila Deshar**: methodology, data curation, validation, formal analysis, supervision, funding acquisition, resources, project administration, writing – review and editing, visualisation, conceptualization.

## Funding

This work was supported by the Department of Environment, Ministry of Agriculture, Forest and Environment, Government of Nepal, and the Nepal Academy of Science and Technology.

## Ethic Statement

This study was conducted in accordance with the ethical guidelines for animal research provided by the Department of National Parks and Wildlife Conservation (DNPWC), Government of Nepal. Fieldwork and sample collection were performed under a research permit (permit number: 176/078/079/DNPWC). As sampling was non‐invasive (fecal collection), no animals were captured, handled, or distressed during the study.

## Conflicts of Interest

The authors declare no conflict of interest.

## Data Availability

The data that support the findings of this study are available from the corresponding author upon reasonable request.
